# Enhancing nurse faculty resilience through self-leadership: guidelines for resource mobilization in dynamic academic environments

**DOI:** 10.3389/fpsyg.2024.1280561

**Published:** 2024-10-29

**Authors:** Vhothusa Edward Matahela, Gisela Hildegard van Rensburg

**Affiliations:** Department of Health Studies, College of Human Sciences, University of South Africa, Pretoria, South Africa

**Keywords:** nurse faculty, resilience, self-leadership, internal sources, nursing education, guidelines, leadership, mental health

## Abstract

The purpose of the study was to develop guidelines for the facilitation of self-leadership in nurse faculty. Of the 12 guidelines developed, this paper discusses the two related to resilience through self-leadership practices, namely: encouraging reliance on internal sources for self-preservation; and strengthening the positive self-image of nurse faculty through management and peer support. An exploratory, sequential mixed-methods design was used to guide the development of guidelines. Enhancing nurse faculty resilience can be achieved by promoting engagement in self-leadership activities. These activities contribute to faculty members’ profound satisfaction, confidence in their knowledge, and overall competence. Resilience is enriched through practicing self-leadership in a supportive work environment and plays a crucial role in adapting to significant changes in the work environment. It has been identified as a key factor that facilitates the ability to persist against struggles and challenges in the workplace. The implementation of higher education reforms in South Africa has brought about increased workload, stress, and uncertainties to an already overwhelmed nurse faculty workforce, consisting of mainly female faculty. It is prudent that a supportive environment that empowers nurse faculty well-being and resilience be facilitated to ensure adaptation to a dynamic and competitive nursing education environment.

## 1 Introduction

Self-leadership, defined as the process by which individuals influence themselves to achieve the self-direction and self-motivation necessary for high performance (Angus et al., 2015; Manz, 1986) has become an essential competency in today’s complex and dynamic work environments. In the realm of higher education, resilience, or the ability to positively adapt to adversity (Masten, 2001), has also emerged as a critical attribute, particularly for nursing faculty, who frequently face high-pressure conditions (Tenschert et al., 2024). Recent literature calls for further research into the interplay between self-leadership and resilience, particularly in nursing education, where rapid institutional reforms pose significant challenges for faculty (Artuch-Garde et al., 2017). As reforms reshape the landscape of higher education, understanding how self-leadership fosters resilience among nurse faculty is increasingly important (Angus et al., 2015).

Self-leadership is grounded in Bandura (1986) social cognitive theory, which suggests that individuals can regulate their thoughts, emotions, and behaviors through self-monitoring and self-reward. Resilience, on the other hand, is rooted in resilience theory, with foundational works by Masten (2001) and Rutter (1987) emphasizing the capacity to recover from adversity. These frameworks have informed various studies on how professionals, including educators, cope with stress and adversity (Manz and Sims, 2001). The self-leadership strategies of goal setting, self-observation, and self-reward have been extensively studied, while resilience theory highlights adaptive responses to persistent stressors in complex environments (Masten, 2001).

However, significant gaps remain in the literature, particularly concerning how nurse faculty utilize self-leadership to build resilience in response to the unique challenges of educational reforms. Most studies, including those by Angus et al. (2015), Manz (1986), and Manz and Sims (2001) focus on general faculty populations, neglecting the specific adversities faced by nurse educators in environments shaped by policy reforms and increased performance expectations. Moreover, while quantitative research on self-leadership and resilience is abundant, there is limited qualitative exploration of nurse faculty’s lived experiences and how these experiences inform their development of self-leadership in the context of ongoing reforms (Matahela, 2023). The methodological emphasis on broad, non-contextual studies fails to capture the nuanced challenges specific to nursing education.

The study seeks to address these gaps by achieving the following objectives: (a) identify the specific self-leadership resources that enable nurse faculty to persevere, endure, and thrive amidst nursing education reforms within NEIs; and (b) explore how nurse faculty develop self-leadership resources that facilitate their sustained commitment to working in NEIs, despite evolving and dynamic working conditions. Thus, the study aims to develop comprehensive guidelines that promote resilience in nurse faculty, ultimately enhancing the quality of teaching and learning in nursing education.

Nurse faculty operate in a dynamic and competitive higher education landscape, wherein they must adapt and innovate to stay ahead in today’s agile world. Numerous studies have highlighted the challenges faced by nurse faculty, which can significantly impact their daily functioning (Reyes et al., 2015). The adversities encountered by nurse faculty include transitioning from a clinical role to an educational role, balancing theory and work-integrated learning, dealing with technological advancements, managing uncivil behaviors, coping with perceived work overload, and navigating the challenges posed by global crises like the Covid-19 pandemic (McDermid et al., 2016; Bagcivan et al., 2015; Ulmen, 2019; Baroudi and Shaya, 2022; Jaimon, 2022; Singh et al., 2020; Ross et al., 2023). Undoubtedly, these situations induce stress, increase instability in the workplace and have a negative impact on the teaching and learning processes in NEIs. Adversity, defined as disruptive events or experiences that can negatively affect psychological and physical well-being (Bonanno, 2004), encompasses various factors such as exposure to threats and violence, working under constant public scrutiny or with uncertainty, increased workloads, and even pressure to produce scholarly work (Adamson et al., 2014).

One often overlooked source of adversity and stress among faculty is the implementation of national policy reforms, primarily due to the uncertainties and ambiguities associated with them (Adamson et al., 2014). This is often the result of a lack of consultation prior to policy reform. Faculty members are at the forefront of implementing these reforms, requiring them to collaborate and align their efforts, even when there may be discrepancies in their understanding of institutional goals and reform expectations (Ganon-Shilon and Schechter, 2019). Adversity in this context often arises because the reform may question or challenge long-established educational practices and the status quo, which may be influenced by faculty’s deeply ingrained educational views (Lockton and Fargason, 2019). Uncertainties and even resistance may arise in terms of the policy content, structure and implementation. These uncertainties could even be a result of unconscious bias and fear of failure.

In recent years, the South African nursing education sector, along with other post-secondary education institutions, has undergone reforms to align with the new higher education qualifications framework and its regulations, aiming to produce a competent nursing workforce to meet current and future healthcare needs (Crowley and Daniels, 2023; Makhanya et al., 2022). Experiences of nurse faculty during the reform implementation process have revealed various adversities arising from perceived incompatibility amongst various legislation instruments, resulting in anxieties amongst faculty (Zwane and Mtshali, 2019). Unpredictable changes can lead to anxiety for nurse faculty, who may need to create meaningful and mindful situations to manage their anxiety. However, changes are not always fully understood or communicated correctly, which can make it difficult for nurse faculty to cope. Uncertainties surrounding funding models and the implementation of transitioning to higher education have also been identified as concerns (Zwane and Mtshali, 2019). Additionally, nurse faculty have faced challenges such as ill-informed expectations about standardized curricula development and delays in finalizing curriculum submission and accreditation pathways (Matlakala and Maritz, 2019). The increased demand for nurse faculty to attain at least a master’s degree in order to meet accreditation requirements (Crowley and Daniels, 2023) has contributed to additional pressure and challenges faced by nurse faculty. The sustained nature of the reforms has also had a negative impact on nurse faculty. As Kerrissey and Edmondson (2023) have posited, when individuals are faced with prolonged ambiguity and challenges, they often exhibit negative reactions as they witness the transformation of their environment. This is because they are uncertain about the future and may feel a sense of loss and/or losing control and may also be concerned about their ability to adapt to the changes. This negativity seems to arise from a yearning for the familiar past, thus reinforcing a sense of disconnection and disorientation from the ongoing changes.

An ongoing sustained period of uncertainty requires the building of resilience amongst the workforce (Kerrissey and Edmondson, 2023). The facilitation of resilience has been shown to mitigate workplace strain and adversity (Jackson et al., 2007). Resilience, defined as an individual’s ability to positively adjust, bounce back, and cope successfully after adversity (Snyder et al., 1991), can be fostered through the nurturing of professional relationships, maintaining positivity, self-awareness, work-life balance, spiritual well-being, and self-reflection (Nickson, 2021). To cultivate these personal strengths, which include stress management competencies, individuals must adopt an optimistic mindset, and persist in pursuing their personal and professional objectives so that they do not succumb to despair in the face of setbacks, crises, and obstacles (Bandura, 1995). Optimism is closely linked to self-leadership and self-regulatory behaviors (Dolbier et al., 2001). Self-regulation encompasses the capacity to control one’s thoughts, emotions, impulses, and performance (Vohs and Baumeister, 2004). Strengthening self-regulation requires the cultivation of various strengths that facilitate the establishment of standards, monitoring of progress, and adaptability (Snyder et al., 1991). Self-regulation may become a preventative measure for many disorders among faculty like anxiety, depression and substance abuse. However, an individual’s capacity for self-regulation can decline over time, necessitating the replenishment of personal resources to prevent lapses in self-control (Baumeister and Vohs, 2016). Therefore, individuals going through uncertainties need to possess self-leadership so that they can maintain the awareness and motivation needed to exert self-control over their thoughts and behavior (London, 2001).

The concept of self-leadership draws from related theories such as self-regulation (Carver and Scheier, 1981), self-control (Thoresen and Mahoney, 1974), and self-management (Manz, 1983), emphasizing the internal control individuals have over their thoughts, motivation, and behavior. The frameworks that provide explanations how individuals can influence their motivation, cognition and behavior are Bandura’s social learning theory and social cognitive theory (Bandura, 1986; Bandura, 1977). Through continuous interaction between individuals and their environment, behavioral self-regulation processes come into play, involving the monitoring of performance levels and the pursuit of self-set goals (Carver and Scheier, 1981). However, environmental disruptions can impact an individual’s self-regulation process and their response to adversity (Manz, 2015). Self-leadership strategies are utilized to bolster the effectiveness of self-regulatory processes, including behavior-focused strategies (self-observation, self-goal setting, and self-reward), natural reward strategies (perceiving and incorporating enjoyable aspects into work tasks), and constructive thought strategies (positive self-talk and visualization of successful performance) (Manz and Neck, 2004; Norris, 2008). According to Krampitz et al. (2023), there is a growing body of research suggesting a significant association between self-leadership and stress management competencies and resilience. Moreover, resilience is considered not only as a capacity but also as a positive psychological state of personal growth. Consequently, it can be inferred that individuals with strong and sound self-leadership competencies are likely to exhibit enhanced resilience (Krampitz et al., 2023).

In order to ensure the resilience and continued growth of nurse faculty amidst adversities, it is imperative to develop strategies that enable their adaptation, fostering an environment of enduring, learning, and rebounding, ultimately contributing to the facilitation of high-quality student learning and teaching. This article aims to examine the mechanisms by which nurse faculty facilitate resilience through engaging in self-leadership practices. Consequently, the study undertakes an investigation of two fundamental questions: (a) What specific self-leadership resources enable nurse faculty to persevere (endure and thrive) in the context of nursing education reforms within a NEI? (b) How do nurse faculty develop the self-leadership resources that facilitate their continued commitment to working in NEIs amid an environment with ever evolving and dynamic working conditions? Thus, the development of comprehensive guidelines aimed at promoting nurse faculty resilience through self-leadership practices becomes essential.

## 2 Materials and method

### 2.1 Design

We conducted an exploratory, sequential mixed methods design study among nurse faculty in two provinces of South Africa. These provinces were similar in terms of governance and development. The overarching focus of this study was to gain insight into the concept of self-leadership among nurse faculty and to generate guidelines that could enhance self-leadership practices. Achieving this objective required initiating an integrative literature review to explore and describe the meaning of self-leadership within the context of a NEI setting with the guidance of Whittemore and Knafl’s (2005) framework. Subsequently, qualitative methods (Polit and Beck, 2021) were employed to delve how the nurse faculty perceived their own self-leadership and ascertain how it could be nurtured. Additionally, descriptive quantitative methods (Gray and Grove, 2021) were utilized to determine nurse faculty’s self-leadership practices. Face validity and the final interpretations of the quantitative data were established by making use of a statistician. Finally, through employing deductive and inductive reasoning, the findings from the various phases of the study were integrated to develop and validate guidelines (Johnstone, 2004; Kredo et al., 2016) aimed at promoting nurse faculty self-leadership. These integrated findings are presented and discussed in a narrative format. In this article, we specifically focus on the integrated findings that underpin two guidelines concerning nurse faculty resilience through self-leadership practices, namely the encouragement of reliance on internal sources and the reinforcement of positive self-image through management and peer support. We elaborate on these findings in the results section.

### 2.2 Participants and procedure

#### 2.2.1 Setting

The study was conducted in 15 NEIs in two provinces of South Africa. These institutions included private and public nursing colleges, as well as universities. The study participants were nurse faculty who were employed at these institutions.

#### 2.2.2 Sources of data

The study utilized data from two distinct populations. Population 1 involved relevant data on academic self-leadership published in English between 2000 and 2019, which was gathered through an integrative literature review. On the other hand, Population 2 consisted of nurse faculty from the selected NEIs who actively participated in the qualitative and quantitative phases of the study. These nurse faculty members were actively engaged in full-time teaching of student nurses for at least a year at NEIs located in urban areas. To avoid overlap, institutions that took part in the qualitative phase were excluded from consideration in the quantitative phase of the study.

#### 2.2.3 Data collection

##### 2.2.3.1 Integrative literature review

An integrative literature review was conducted to collect data from various theoretical and empirical sources published between 2000 and 2019. Multiple computer-assisted search engines were utilized, guided by specific keywords.

##### 2.2.3.2 Qualitative phase

During this phase, which was completed in 2019, four focus group interviews with nurse faculty were conducted at their workplace. Four purposefully selected focus group interviews were conducted, each consisting of five to eight nurse faculty. Each focus group was made up of at least 10 nurse faculty, all of whom were female. These interviews followed a semi-structured format, using prepared questions and areas for in-depth probing. Participants were provided with the interview guide in advance. The interviews took place in natural settings with permissions and informed consent. Various communication techniques were employed to gather in-depth data. These techniques included the interviewer using reflection, silence, nodding, and active listening. The interviews were conducted were audio-recorded with the consent of all participants. Additionally, field notes were taken throughout the interviews, allowing for the capture of essential non-verbal cues and subtle expressions, thereby enriching the data gathered during the sessions. Transcriptions of the conducted interviews, along with the accompanying field notes, were thoroughly reviewed to gain a deeper understanding of the collected data.

##### 2.2.3.3 Quantitative phase

To collect quantitative data on nurse faculty’s self-leadership practices, a structured questionnaire was designed. This questionnaire encompassed 78 items and was based on a seven-point Likert scale, meticulously integrating themes derived from the literature review and qualitative phase, as well as constructs from Manz’s (1986) self-leadership theory. The questionnaire was administered in English, featuring a combination of closed-ended and open-ended items. It consisted of four sections: socio-demographic data (Section A, 7 items), perceptions of nurse faculty’s self-leadership concept and constructs (Section B, 29 items), nurse faculty’s self-leadership practices (Section C, 33 items), and the role of motivation in nurse faculty’s self-leadership (Section D, 9 items). The questionnaire underwent a pre-testing process with 16 participants who were not part of the main study. Following the successful pre-test, a total of 443 nurse faculty members from 15 different NEIs were approached and invited to participate in the study. Questionnaires were either hand-delivered or distributed through an electronic platform (SurveyMonkey). In most institutions, the first author or the research coordinator personally collected the completed questionnaires. Following collection, stringent measures were implemented to ensure the security and confidentiality of the data, and accessibility was limited to the researchers. A total of 252 (67%) nurse faculty filled in the hand-delivered questionnaires and 19% utilized the electronic platform (*n* = 13), resulting in a total of 265 nurse faculty participants, who comprised of 250 (94%) females and 15 (Bandura, 1986) males.

##### 2.2.3.4 Data integration

Both qualitative and quantitative data were collected and analysed to understand the research topic comprehensively. The qualitative data, which typically involved thematic analysis, and the quantitative data, which involved statistical analysis, were integrated into a single narrative (Yin, 2016). This integration was accomplished by describing the findings from both data sets in a single report, ensuring that the insights from the qualitative data complemented the statistical trends and outcomes from the quantitative data.

The combination of these findings enabled a thorough discussion, highlighting patterns, discrepancies, and areas of convergence between the two data sources (Yin, 2016). From this integrated analysis, guidelines were subsequently developed. These guidelines were based on the integrated evidence derived from both qualitative experiences and quantitative measures, ensuring that they were grounded in a robust and balanced understanding of the research findings.

### 2.3 Ethical considerations

The study received ethical approval from the University’s Research Ethics committee (REC-012714-039) and obtained necessary permissions from relevant research bodies, health authorities in NEIs, and provincial health departments. All research procedures strictly adhered to the ethical standards outlined in the Helsinki Declaration of 1975 (revised in 2013). Participants were fully informed about the study’s objectives and provided their informed consent to participate. They were assured of confidentiality, and it was emphasized that they could withdraw from the study at any point without facing any negative consequences.

### 2.4 Data analysis

#### 2.4.1 Integrative literature review

Miles and Huberman’s thematic data analysis technique (Whittemore and Knafl, 2005) was utilized. This involved data reduction, data display, data comparison, conclusion drawing, and verification. The reviewed studies described self-leadership in nurse faculty positively, indicating that engaging in self-leadership practices improved academic performance across various educational settings. However, there was a lack of specific literature on nurse faculty’s self-leadership in NEIs, leaving the phenomenon incompletely defined, thus needing further exploration by subsequent methods.

#### 2.4.2 Qualitative analysis

Verbatim transcripts and field notes from semi-structured focus group interviews were independently analysed using Tesch’s thematic analysis protocol (Creswell and Creswell, 2018). The first author and an independent co-coder conducted the analysis. Categories and subcategories were identified independently and then discussed in a consensus meeting. Further discussions between the authors refined the analysis, identifying patterns and gaps, and organizing the data into logical themes and subthemes. The resulting themes included perceptions of nurse faculty self-leadership, engagement in self-leadership practices, motivational factors influencing self-leadership, and facilitating self-leadership among nurse faculty.

#### 2.4.3 Quantitative analysis

The raw quantitative data were coded and captured in a Microsoft Excel spreadsheet. A statistician used SPSS Version 25 to analyse the data descriptively. To validate the questionnaire constructs, an exploratory factor analysis (EFA) was performed on the construct responses. The analysis, following the maximum likelihood method and a varimax rotation, assessed whether the individual questions aligned with the intended constructs. Validity testing of the motivation construct was followed by item analysis to assess the construct’s reliability using Cronbach’s alpha coefficient, confirming the consistent measurement of relevant items for each construct in the questionnaire (Polit and Beck, 2021).

#### 2.4.4 Guidelines

Based on the integration of data and literature, concluding statements were formulated and resulted in the development of 12 guidelines that aim to facilitate self-leadership among nursing faculty. We invited expert reviewers to assess the guidelines for clarity, comprehensiveness, applicability, adaptability, credibility, and validity, following criteria outlined by Chinn and colleagues (Chinn et al., 2021). In this article, we focus on two of the guidelines, specifically addressing resilience through self-leadership practices.

#### 2.4.5 Rigor

The integrative literature review incorporated diverse empirical and theoretical sources while adhering to the University’s Policy for Copyright Infringement and Plagiarism to maintain academic integrity. Various stages of research review were followed, including well-defined search strategies that encompassed manual hand searching, research journals, networking, and computer-based searches.

For the qualitative phase, Lincoln and Guba’s (1985) criteria of trustworthiness were applied to ensure credibility, transferability, dependability, authenticity, and confirmability. Credibility was achieved through accurate data description and presentation, allowing other nurse academics to relate to the experiences. Prolonged engagement, triangulation, and member checking were employed to enhance trustworthiness.

In the quantitative phase, content validity, face validity, and construct validity were ensured. Measures of validity and reliability were used to establish internal and external validity. Content validity was accomplished through an integrative literature review, discussions, and consultations. Face validity was established through the assessment of the questionnaire by a statistician and independent nurse faculty. Construct validity was supported by incorporating the self-leadership theoretical framework and integrative literature review. The Cronbach alpha coefficient ranged between 0.6 and 0.8, indicating satisfactory reliability. Pre-testing of the questionnaire further contributed to reliability.

## 3 Results

The study integrated both qualitative and quantitative data, thus, presenting a comprehensive description of the findings from both data sets within a single report (Fetters et al., 2013). The three themes that emerged from the integrated data (qualitative and quantitative) were (a) taking ownership of one’s self-leadership in self-reflection on one’s behavior; (b) motivational factors in nurse faculty self-leadership; and (c) facilitation of self-leadership in nurse faculty. In this article, we focus on the theme *Facilitation of self-leadership in nurse faculty*, which addresses promotion of nurse faculty resilience through self-leadership practices. If self-leadership practices can help employees to manage adversity (Lovelace et al., 2007), then there is need for understanding how individuals and NEIs can proactively support nurse faculty to develop the resilience needed to manage the stressful demands of today’s academic work environment. The theme had four sub-themes (discussed below).

### 3.1 Exercising self-control to sustain effective teaching presence

In response to an open-ended question, 33 (12%) participants expressed their perception of possessing self-control by associating the concept of “self-leadership” with self-discipline and self-control. They identified certain situations where nurse faculty may exhibit a loss of self-control, including the inability to manage emotions, the inclination to react emotionally or become upset when provoked in class, and the struggle to maintain composure, even considering shouting at students during lessons.

Participants viewed self-leadership as embodying self-control and self-discipline, enabling them to prioritize and concentrate on their duties at the NEI. They stressed the significance of nurse faculty “staying present” during challenging encounters as part of their adapting to changes and in developing resilience. Participants emphasized the importance of managing situations that could lead to a loss of self-control, advocating for mindfulness and not succumbing to the circumstances, as quoted:

*“There are so many situations that can make one to lose self-control. But the main thing is how to manage those situations. Be mindful and do not flow with a situation that makes you to lose self-control.”*

Furthermore, participants highlighted the need to remain focused and seek support from acquaintances who may be willing to make small adjustments to assist:

*“So those are situations that can derail your focus, but you do not have to lose self-control. Know that you are not alone, you are with other people. Be accommodative and you see how others are willing to be able to help sort situations like that out.”*

The citations acknowledge that stressful situations can divert focus; however, nurse faculty should avoid losing self-control and instead invest time to accommodate and listen to others’ perspectives for resolving such situations. Therefore, in the face of adversity, nurse faculty must possess personal resources to replenish themselves and maintain self-control. These resources include self-awareness of motivations, reflection on goals and past achievements, emotional regulation, and evaluating the effectiveness of the coping mechanisms used (Baker et al., 2021). Reflecting on both successes and challenges strengthens resilience skills and instils confidence for future utilization. Awareness and cultivation of personal strengths allow individuals to harness their best attributes, fostering overall well-being when present in the situation and employing character strengths (Niemiec, 2020). Being present involves establishing connections with students, their learning, subject matter, and with oneself (Rodgers and Raider-Roth, 2006). By cultivating presence, educators become fully engaged in teaching and learning activities, enabling them to better recognize, interpret, and respond appropriately to what is happening (Doornich and Lynch, 2024). Therefore, by leveraging their character strengths, nurse faculty express gratitude, acknowledge their experiences, and remain fully attentive to themselves, students, and learning, enabling skillful and compassionate teaching despite the challenging environment.

### 3.2 Promoting self-care through positive role modelling

Role modelling plays a pivotal role in promoting self-leadership and is particularly significant in the context of nurse faculty. When asked how they exemplified self-leadership through role modelling for students, participants provided the following responses: 120 participants (45%) demonstrated it by consistently exhibiting professional behavior and attire; 45 participants (17%) emphasized the importance of respecting time and being punctual; 43 participants (16%) highlighted the significance of showing respect to students; and 31 participants (6%) emphasized adherence to the prescribed dress code. Conversely, participants expressed their demonstration of role modelling for their colleagues through the following practices: 61 participants (23%) underscored the importance of demonstrating respect; 52 participants (19.6%) emphasized the maintenance of professional relationships; 20 participants (7.5%) emphasized effective communication; and 19 participants (7%) highlighted the significance of punctuality. Notably, participants expressed the expectation that their managers would serve as role models, as this could inadvertently promote self-leadership among nurse faculty.

Participants expressed their belief in the importance of serving as role models for both colleagues and students by embodying the desired behaviors, values, and professional image of the nursing profession. They viewed themselves as leaders, offering a positive example for others to follow, even during times of change and reforms. One participant articulated this viewpoint, stating:

*“With all these changes that are currently happening in nursing education, I need to be flexible enough, because if I am not, I will become resistant to change, and will become burnt out and definitely call it quits… so I need to ensure I deal with issues with the right mindset and take care of myself…”*

Given the demanding nature of their roles, nurse faculty face significant stress and burnout, particularly during periods of reform implementation. This stress can sometimes hinder their ability to fulfil their role-modelling responsibilities. Empirical evidence has indicated that regular engagement in self-care practices can mitigate the impact of high stress levels among faculty and serve as effective coping strategies during challenging times (Kutsyuruba et al., 2021). Furthermore, emerging research suggests that mindfulness, self-care practices, and overall well-being can act as resilience-building strategies, protecting nurses from the adverse effects of workplace stress (O’Malley et al., 2023; Matahela and van Rensburg, 2023). Therefore, it is imperative for nurse faculty to acquire mindfulness techniques as self-care strategies, as this can enhance relaxation, strengthen coping mechanisms, and help to reduce stress.

By cultivating greater self-awareness through mindfulness practices, nurse faculty can prioritize their own well-being and self-care behavior, thereby positively influencing their role modelling through teaching and learning activities (Matahela and van Rensburg, 2023). Active engagement in self-care activities can strengthen mindfulness and foster the faculty’s awareness of the profound impact their actions and behaviors have on those who seek their guidance (Wong, 2004). Self-care activities encompass a range of practices, including engaging in spiritual introspection and contemplation, practicing mindful breathing techniques, and exploring artistic pursuits like painting, sculpture, and pottery (Harmon et al., 2018). Additionally, these activities may involve participating in meditation and yoga classes, taking part in retreat activities, maintaining a reflective journal, and engaging in group discussions (Matahela and van Rensburg, 2023).

### 3.3 Enhancing nurse faculty resilience through management and peer support

In order to sustain nurse faculty resilience, the provision of management and peer support plays a crucial role, as indicated by the participants. Several situations were identified where self-leadership could be facilitated through such support. These included recognizing achievements, assisting nurse faculty to navigate complex emotional and psychological situations within the institutional context, and ensuring the availability of teaching and learning resources in both classroom and clinical settings.

While participants acknowledged the potential of management support in fostering their self-leadership, they rated the intrinsic motivation construct as more significant. This finding suggests that self-leaders rely primarily on their internal motivation to perform effectively, rather than depending on their peers or managers to enhance their performance or job-related aspects.

Among the 264 participants (1 missing), only 80 (30%) strongly believed that their colleagues valued and supported their work, while 128 (48%) strongly believed that institutional management should offer support to enhance their performance. The desired areas of support from management included establishing a respectful and equitable environment and offering guidance for challenging tasks. Participants expressed the need for peer support, particularly during times of reform, highlighting the lack of spontaneous support within their work environment, as quoted:

*“One thing that nurses cannot do well is to support each other. In my opinion, support should be a spontaneous thing. But no, we let each other drown in the deep end….”*

*“I think two heads are better than one, we need to support each other and to boost the group moral and … then the students will also see that we are working as a team and that – then they would also like have more respect for us or see us as leaders.”*

Participants expected institutional managers to mobilize resources and leverage their expertise, knowledge, and skills to promote nurse faculty resilience. One participant emphasized the importance of supportive working conditions, stating that individual efforts alone might not be sufficient to overcome the overwhelming workload:

*“We need to be assisted with resources… I can try my level best as an individual, but I can still drown because I am overwhelmed with the load of work that is on my shoulder, so my employer must ensure that working conditions are conducive.”*

Based on the citations, it is evident that participants did not experience collegiality or a supportive culture of quality within their NEIs. Thapa et al. (2021) argue that institutional and collegial support are vital resources for nurses facing difficult times, as these facilitate recovery and overall well-being. Nurse faculty in possession of self-leadership skills are known to provide social networks and support to their colleagues (Matahela and van Rensburg, 2021). This support is enabled by effective communication that strengthens interpersonal processes and develops into meaningful relationships that foster resilience, emotion regulation, and better understanding of perceived threats (Finstad et al., 2021). Resilience is also built through investing time in recognizing available resources and establishing trustworthy relationships with colleagues and managers who have themselves shown personal resilience before (McDermid et al., 2016). This highlights the importance of self-leadership, as these colleagues serve as role models in demonstrating resilience. Therefore, institutional leaders must possess self-leadership qualities, and strive to be resilient, adaptable and agile individuals (Green-Wilson et al., 2022). By embodying these attributes, leaders can effectively navigate change and promptly adapt their behaviors, strategies, and ideas to guide faculty to cope and to respond to the evolving demands of both the dynamic environment and the future.

It is apparent that while self-leadership is an inherently an individual process, maintaining its practices and building resilience over time can be challenging without external support. Therefore, both the colleagues and institutions have a responsibility to create supportive and positive work environments (Park and Byon, 2024). By offering emotional support, both these supportive resources can foster resilience and mitigate nurse faculty burnout.

### 3.4 Enhancing nurse faculty’s resilience through self-leadership strategies training

Self-leadership in nurse faculty can be facilitated by various factors, including professional development and training in self-leadership strategies (Griffiths, 2023). Participants identified a need for self-leadership training-related programmes that could empower them with the resilience required to cope with the stress that comes with the implementation of reforms. Among the 150 participants who provided responses regarding examples of training programmes that could foster self-leadership, the majority of 121 (81%) listed training programs with innovative teaching and discipline-specific courses, whilst 32 (21%) of them mentioned training programs focused on self-awareness and emotional intelligence, and self-leadership strategies.

Participants highlighted the significance of institutions offering targeted training programs that address resilience amid reforms for nurse faculty. They emphasized the value of additional support, such as regular check-ins with faculty, access to resources and information on self-care, resilience strategies, and opportunities for collaboration and debriefing, all of which play a vital role in fostering resilience in the face of adversity (Parayil-Pezard, 2020). However, participants also suggested that individual nurse faculty should take the initiative to seek self-leadership training in programs related to self-awareness and emotional intelligence, rather than solely relying on institutional offerings. Among the suggested training programs to facilitate self-leadership in nurse faculty, mindfulness training was highlighted as a valuable tool, as indicated:

*“I found the course to be extremely valuable in enhancing my self-awareness and resilience as an educator. It helped me recognize both my weaknesses and strengths, allowing me to view that inner critical voice as just a voice, rather than reality.”*

*“As educators, we often experience fear or worry in the classroom. By consistently addressing these thoughts and acknowledging that they may stem from feelings of inadequacy, we can confront them and work through them. For example, if I succumb to thoughts of inadequacy or doubt my ability to complete my PhD, I remind myself to take things one step at a time. It’s like eating an elephant – I break it down into manageable pieces and see where each step takes me. This approach has become my life goal – to tackle challenges gradually and make progress.”*

By incorporating mindfulness into self-leadership training for nurse faculty, they can cultivate improved emotional awareness, enhance their ability to regulate emotions, and experience a greater sense of calmness, relaxation, and self-acceptance (Parayil-Pezard, 2020). This type of training should guide nurse faculty in developing a vision for engaging in self-care practices and planning personalized lifestyle behaviors to enhance optimal functioning and fulfilment, encompassing overall well-being (Parayil-Pezard, 2020).

Thus, nurse faculty’s resilience can be achieved through a holistic approach to well-being. Therefore, it is recommended that nurse faculty actively engage in institutional wellness programs that address holistic dimensions encompassing environmental, physical, social, emotional, spiritual, and intellectual realms. This participation in integrated wellness programs will contribute to enhancing their overall well-being and functioning, and resilience.

## 4 Discussion

Utilizing integrated data, this study aimed to develop guidelines designed to enhance the practice of self-leadership among nurse faculty. Following the development of these guidelines, a validation process was conducted with the participation of field experts possessing diverse backgrounds in nursing education, practice, leadership, and policy development. This article presents the guidelines which were derived from an integration of empirical evidence and existing literature, alongside the conclusive statements documented in Table 1. Moreover, recommendations are provided to aid in the formulation of these guidelines.

**Table 1:** Concluding statements synthesized from integrated findings.

Theme	Category	Concluding statement	Guideline
Facilitation of self-leadership in nurse faculty	Exercising self-control to sustain effective teaching presence.	Individuals possess the capacity exert self-control (self-regulation) over their thoughts, emotions, motivations, and behaviors.	Encouraging reliance on internal sources for self-preservation
		The high emotional demand and stress that comes with teaching should be managed.	
		Exercising self-control enables nurse faculty to recognize and address emotions that may disrupt the teaching and learning process, as well as their interpersonal relationships.	
	Promoting self-care through positive role modelling.	Nurse faculty are cognizant of their influential role in shaping aware that they play an influential role in the professional socialization and the developmental journey of students.	
		Practicing mindfulness cultivates focused attention on contexts and activities, while maintaining an accepting and non-judgmental mindset.	
		Practicing holistic self-care practices and adoption of wellness-focused behaviors facilitates the achievement of optimal individual functioning and fulfilment.	
	Enhancing nurse faculty resilience through management and peer support.	Nurse faculty experience enhanced motivation and emotional stimulation to sustain their teaching engagement through the provision of peer support.	Strengthening the positive self-image of the nurse faculty through management and peer support
		Nurse faculty expect recognition, respect, fair treatment, and support when working in challenging contexts.	
		Access to expertise and resources enable nurse faculty self-leadership practices	
	Enhancing nurse faculty’s resilience self-leadership strategies training.	Institutional investment in the development of faculty self-leadership strategies promotes adapting and enhancement of their self-leadership skills and resilience, empowering them to build resilience by embracing and leveraging of own strengths.	
		Leadership programs that foster an authentic and supportive work environment could facilitate a work climate resilience.	

### 4.1 Guideline 1: encouraging reliance on internal sources for self-preservation

Self-leadership fosters a mindset that emphasizes reliance on internal resources for self-preservation, thus encouraging individuals to tap into their own strengths and skill to navigate challenges and protect their well-being (Harunavamwe et al., 2020). When faced with uncertainty and perceived obstacles, individuals will then be able to exercise self-control and draw upon their own inner reservoirs of resilience, determination, and problem-solving abilities to overcome these obstacles and thrive in challenging situations (Dhiman, 2023).

The purpose of this guideline is to encourage nurse faculty engagement in self-leadership practices through self-control and role modelling. This enables them to stay motivated and maintain a clear focus on their established goals and uphold their personally chosen beliefs, even during a period of uncertainty. Nursing faculty demonstrate self-control and role modelling by:

- Cultivating self-awareness and self-reflection to maintain internal control over their emotions, thoughts, and behaviors, influencing the teaching-learning process and enabling informed decision-making when engaging with students and colleagues.
- Fostering supportive and nurturing relationships with students through caring behaviors, trust, shared power, and respect for their learning needs.
- Maintaining self-discipline, including preparation, effective teaching skills, sensitivity to student needs, and respectful treatment.
- Building credibility by showcasing consistency and fairness across various activities and creating a mutual understanding with students regarding classroom rules, marking criteria, monitoring student behaviors, and ensuring the fulfilment of commitments.
- Cultivating mindfulness by observing emotional awareness and regulation, controlling thoughts and impulses, resisting temptations, and breaking bad habits.
- Role modelling positive behaviors through engaging in self-care practices.

The NEI supports nurse faculty by proactively implementing strategies that address psychological well-being (Gilar-Corbi et al., 2024) in nurse faculty, reducing emotional exhaustion, and work disengagement, which may negatively affect self-leadership.

### 4.2 Guideline 2: strengthening the positive self-image of the nurse faculty through management and peer support

Self-leadership strategies, particularly the constructive thought processes, promote positive thinking by addressing maladaptive beliefs and assumptions, minimizing negative self-talk, and fostering a positive self-image (Neck and Houghton, 2006). Although these strategies are intrinsically driven, external factors such as self-leadership skills training, collegial and management support can influence their implementation (Stewart et al., 2019). Therefore, the institution through its managers and fellow colleagues facilitates faculty self-leadership by providing a healthy working environment that fosters nurse faculty self-worth and a positive self-image.

Thus, the purpose of this guideline is to ensure that nurse faculty are equipped with the requisite expertise and essential resources to effectively practice self-leadership, through supportive interventions from their colleagues and institutional managers. Colleagues support fellow nurse faculty to improve self-image by:

- Cultivating supportive, collegial relationships among faculty members to enhance understanding of roles, concerns related to their careers, and interpersonal dynamics, ultimately boosting self-confidence.
- Fostering a trusting, collaborative environment that mitigates the negative impact of stress in the academic setting.
- Participating in peer learning communities to generate and implement action plans for adapting to uncertain and ambiguous work environments.
- Offering mindful and authentic feedback to colleagues within a psychologically safe climate, supporting their emotional adjustment and social integration.

The NEI supports nurse faculty by:

- Implementing programmes aimed at promoting self-leadership through supporting the nurse faculty’s work-life balance and manageable workloads as defined through engagement and input from the nurse faculty. Offering resilience-building and emotion-regulating programmes such as self-leadership skills, mindfulness and resilience training to counter the stress and challenges faced by nurse faculty. These programs should also incorporate leadership development to foster self-leadership practices within a supportive organizational environment (Kumar et al., 2024).
- Investing in and offering institutional wellness programs that address the holistic dimensions of nurse faculty wellness, functioning and resilience.
- Undergoing self-leadership training and emotional intelligence leadership skills programs, to equip them in creating an authentic and nurturing environment that facilitates a positive empowerment process, promoting the well-being of nurse faculty.
- Utilizing leadership styles that elicit rapid decision-making and an adaptive response (Saleem et al., 2023) to facilitate nurse faculty creativity, innovation and resilience.

## 5 Conclusion

The implementation of reforms in nursing education has been marked by uncertainty and ambiguity in the nursing sector, particularly in NEIs. As key implementers of the reforms, nurse faculty have expressed feelings of anxiety, stress and burnout, which are manifestations of depletion of the personal resources needed for the sustenance of self-control, self-regulation, and resilience. In this study, in which the participants were mainly female faculty, it became clear that there was a need for nurse faculty to be supported so that they can have the confidence to continue providing quality teaching and learning to students in an era characterized by uncertainty and ambiguity. Self-leadership was found to be a strategy that could be employed to facilitate optimal functioning and resilience of nurse faculty in a dynamic, uncertain and constantly evolving environment. Nurse faculty resilience can be nurtured through employing internal sources such as self-control and self-care practices; and strengthening faculty self-image through collegial and management support, as well as through resilience-building training programmes. These resources could enable nurse faculty to persevere and remain committed to working in NEIs in an ever evolving and dynamic work environment.

## Data Availability

The original contributions presented in the study are included in the article/supplementary material, further inquiries can be directed to the corresponding author.
